# Positive correlation of airway resistance and serum asymmetric dimethylarginine (ADMA) in bronchial asthma patients lacking evidence for systemic inflammation

**DOI:** 10.1186/s13223-017-0226-5

**Published:** 2018-01-03

**Authors:** Gabor Tajti, Csaba Papp, Laszlo Kardos, Sandor Keki, Krisztian Pak, Magdolna Emma Szilasi, Rudolf Gesztelyi, Angela Mikaczo, Andrea Fodor, Maria Szilasi, Judit Zsuga

**Affiliations:** 1 Department of Health Systems Management and Quality Management for Health Care, Faculty of Public Health, University of Debrecen, Nagyerdei krt. 98, Debrecen, 4032 Hungary; 2 Institute of Clinical Pharmacology, Infectious Diseases and Allergology, Kenezy Gyula Teaching County Hospital and Outpatient Clinic, Bartok Bela ut 2-26, Debrecen, 4031 Hungary; 3 Department of Applied Chemistry, Faculty of Science and Technology, University of Debrecen, Egyetem ter 1, Debrecen, 4032 Hungary; 4 Department of Pharmacology and Pharmacotherapy, Faculty of Medicine, University of Debrecen, Nagyerdei krt 98, Debrecen, 4032 Hungary; 5 Department of Pulmonology, Faculty of Medicine, University of Debrecen, Nagyerdei krt. 98, Debrecen, 4032 Hungary

**Keywords:** ADMA, Airway resistance, Bronchial asthma, SGRQ, Whole-body plethysmography

## Abstract

**Background:**

Contribution of nitric-oxide (NO) pathway to the pathogenesis of bronchial asthma (asthma) is ambiguous as NO may confer both protective and detrimental effects depending on the NO synthase (NOS) isoforms, tissue compartments and underlying pathological conditions (e.g. systemic inflammation). Asymmetric dimethylarginine (ADMA) is an endogenous inhibitor and uncoupler of NOS with distinct selectivity for NOS isoforms. In a cross-sectional study, we assessed whether ADMA is an independent predictor of airway resistance (R_aw_) in therapy-controlled asthma.

**Methods:**

154 therapy-controlled asthma patients were recruited. ADMA, symmetric dimethylarginine and arginine were quantitated by HPLC with fluorescent detection. Pulmonary function test was done using whole-body plethysmography, quality of life via St. George’s Respiratory questionnaire (SGRQ). Multiple linear regression was used to identify independent determinants of R_aw_. The final model was stratified based on therapy control.

**Results:**

Evidence for systemic inflammation indicated by CRP and procalcitonin was lacking in our sample. Log R_aw_ showed significant positive correlation with log ADMA in the whole data set and well-controlled but not in the not well-controlled stratum (Spearman correlation coefficients: 0.27, p < 0.001; 0.30, p < 0.001; 0.12, p = 0.51 respectively). This relationship remained significant after adjusting for confounders by multiple linear regression (β = 0.22, CI 0.054, 0.383 p = 0.01). FEF 25–75% % predicted and SGRQ Total score showed significant negative while SGRQ Activity score showed significant positive correlation with R_aw_ in the final model.

**Conclusions:**

Positive correlation between R_aw_ and ADMA in the absence of systemic inflammation implies that higher ADMA has detrimental effect on NO homeostasis and can contribute to a poor outcome in asthma.

## Background

Bronchial asthma (hereinafter referred to as asthma) has high socioeconomic impact stemming from premature morbidity, poor quality of life, significant healthcare utilization and loss of work productivity [1, 2]. The key pathomechanistic properties of asthma are the presence of chronic inflammation in the lower respiratory tract and consequent airflow limitation manifested as dyspnea (predominantly in the form of recurrent bouts) [3, 4]. Contribution of the nitric oxide (NO) pathway to the evolution of inflammation in asthma has long been proposed, nonetheless the net effect of altered NO homeostasis in the inflammatory state characteristic of asthma is yet to be elucidated.

The source of controversy is that, similar to other tissues like vasculature [5, 6], NO may confer both protective and detrimental effects that depends on the activity of different NO synthase (NOS) isoforms, on the affected tissue compartment and on some underlying conditions [7]. Of the three NOS isoforms (each expressed in the lung), endothelial NOS (eNOS) and neuronal NOS (nNOS) are Ca-calmodulin dependent constitutive enzymes that liberate low (ranging from femto- to picomolar) concentrations of NO within seconds of receptor activation. The eNOS, apart from vasculature, is chiefly localized in the bronchial epithelium and type II alveolar cells [8], and NO released by this isoform leads to broncho- and vasodilation. The nNOS is chiefly located in peripheral nerves innervating bronchial smooth muscle and submucosal secretory glands. Density of innervation decreases from trachea to smaller bronchi conferring reduced NO-mediated neural bronchodilation in smaller airways [9]. The third isoform is the inducible NOS (iNOS) that, albeit continuously expressed in lung epithelial cells [10], is chiefly present upon its induction by pro-inflammatory cytokines. These latter molecules activate the nuclear transcription factor NF-κB that leads to iNOS expression and thereby sustained high release of NO (in nanomolar concentration) over the course of hours to days [8]. Based on the results of preclinical and clinical studies it seems that iNOS may be produced by alveolar type II epithelial cells, lung fibroblasts, airway and vascular smooth muscle cells, endothelial cells, mast cells and neutrophils [8, 11], and its expression is inhibited by glucocorticoids [12]. Under conditions of inflammation, NO, synthetized by iNOS, and superoxide anion, formed by activated macrophages and neutrophils, react to form peroxynitrite and subsequent inflammatory cell recruitment, airway constriction and remodeling [13]. Change in the level of all three isoforms in distinct compartments has been described in asthma [7]. In asthma patients, increased expression of iNOS was described in airway epithelium [7, 14, 15], while decline in activity of constitutive isoforms was also observed [16]. Furthermore, l-arginine or tetrahydrobiopterin depletion may cause uncoupling of all three NOS isoforms, switching the enzyme’s function to produce superoxide instead of NO [11, 17, 18].

**Fig. 1:**
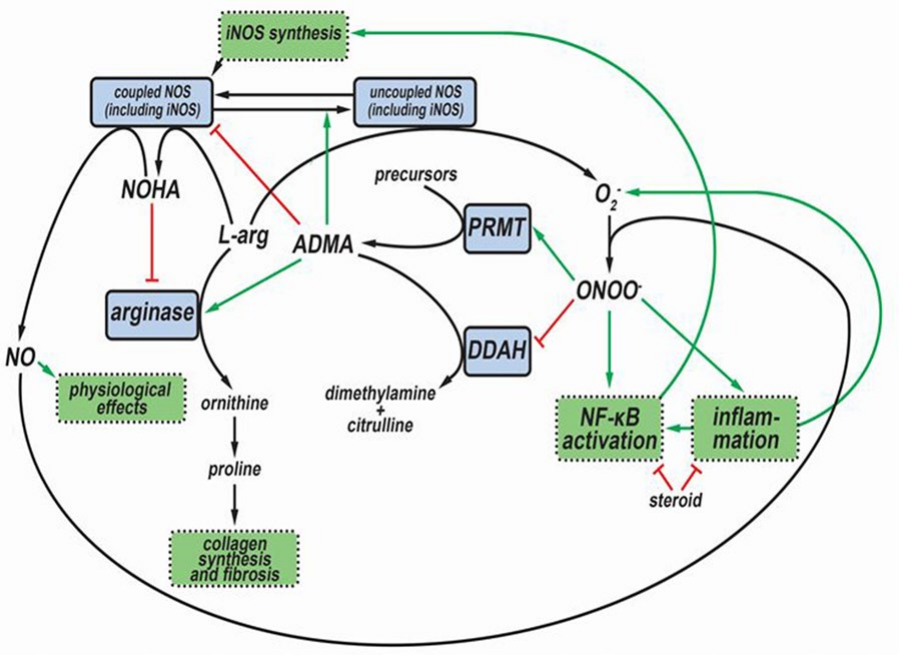
Intertwining of asymmetric dimethylarginine (ADMA) with the nitric-oxide (NO) homeostasis in inflammatory airway diseases. Nitric oxide (NO) is formed from l-arginine through *N*-hydroxyarginine (NOHA) by NO-synthase enzymes (NOS) including the inducible one (iNOS). In bronchial asthma, nuclear transcription factor NF-κB, activated by inflammatory cytokines, increases the expression of iNOS. This augmented expression promotes formation of NO, which compound, by reacting with superoxide anion (O_2_^−^) common under inflammatory conditions, provides peroxynitrite anion (ONOO^−^) and eventually results in nitrosative stress. This stress increases the production of asymmetric dimethylarginine (ADMA) by enhancing the expression of protein methyltransferase enzymes (PRMT), responsible for ADMA formation, and by decreasing the expression of dimethylargininase (DDAH), which participates in the elimination of ADMA. ADMA is an endogenous inhibitor and, in addition, a natural uncoupler of all NOS isoforms. Uncoupling of NOS leads to the production of O_2_^−^ instead of NO. Thus, elevated ADMA level, on one hand, decreases NO production, and, on the other hand, increases levels of oxidative and nitrosative agents via uncoupling NOS. Higher ADMA level also results in enhanced arginase activity, thereby contributing to collagen synthesis and (possibly) to the evolution of a (reversible) lung fibrosis. Corticosteroid therapy can prevent the iNOS-related processes elicited by inflammation (via inhibiting inflammatory processes and directly inhibiting the NF-κB expression) that leads to decreased iNOS expression and consequently lower production of NO, ADMA and oxidative/nitrosative agents. Green arrows: activation, enhancement; red lines: inhibition; black arrows: transformation, metabolic connection

Asymmetric dimethylarginine (ADMA), a metabolite of protein turnover throughout the body, is considered as a significant factor in NO homeostasis that may interfere with several processes related to the evolution of inflammatory airway diseases. ADMA is viewed as an endogenous competitive inhibitor of NOS showing higher selectivity for the constitutive isoforms [11]. In addition, animal studies showed that ADMA is a natural uncoupler of all the three NOS isoforms leading to increased superoxide formation and oxidative as well as nitrosative stress [19]. ADMA may also compete with l-arginine for intracellular transport, thus it may limit l-arginine’s availability as a substrate for NOS [20] and thereby contribute to intracellular l-arginine depletion. Furthermore, exogenous ADMA per se was shown to cause airway hyperresponsiveness, to increase collagen formation and to induce reversible fibrosis in mice [19]. This latter effect may be due to ADMA’s ability to enhance the activity of arginase [20]. While lung (more specifically: airway epithelium) has been shown to be a major source of ADMA [11, 20], conflicting reports have emerged related to the systemic ADMA concentration in asthma [21] with reports describing both higher [22] and normal/low-normal [23] serum levels (Fig. 1).

Starting from the above-mentioned findings, we set out to elucidate whether ADMA is a risk or protective factor in the inflammatory state characteristic of asthma, by assessing the relationship of ADMA with lung function parameters descriptive of airflow limitation.

## Methods

### Study design and protocol

The study was prepared in line with the STROBE statement for cross-sectional studies [24]. Every patient was invited to participate in our study who visited the outpatient unit of the Department of Pulmonology (University of Debrecen) between September 1 2012 and October 15 2013 for the management of chronic airway inflammatory diseases including asthma, chronic obstructive pulmonary disease (COPD), asthma-COPD overlap syndrome (ACOS) and allergic rhinitis (AR). Exclusion criteria were every acute inflammatory disease over the preceding 1 month and malignancies or benign tumors in the history. Overall 319 patients were recruited (asthma: n = 167, COPD: n = 74, ACOS: n = 21 and AR: n = 57). Data of patients with asthma were included in the current analysis.

For the present study, those patients who were already diagnosed with asthma at the time of recruitment and underwent whole-body plethysmography during the recruitment period were involved (n = 154). Accordingly, all these patients participated in a control-based asthma management program complying with GINA as well as the relevant Hungarian practice guidelines [25, 26]. Thus, treatment at the time of inclusion was provided as clinically warranted. Defined daily dose of inhaled corticosteroids was determined to allow comparisons across treatment regimens [27]. R_aw_ is reflective of changes in alveolar pressure over changes in flow as it is highly dependent on state of airways, thus it is an appropriate parameter for quantifying airflow limitation [28]. (The most important parameters determining the state of airways are length and (average) diameter. If we want to characterize the airways with extensive (additive) properties only, these are: length, surface and volume [28]). Demographic and anthropometric data were acquired and body mass index was also calculated. Furthermore, history of smoking, diabetes, dyslipidemia and hypertension were also obtained. Cumulative measure of smoking exposure was characterized with pack-years (computed in a way that both past smoking and current smoking exposure were accounted for). Disease-specific quality of life was also assessed with the use of the official Hungarian version of St. George’s Respiratory questionnaire (SGRQ) [29] after obtaining written permission from its proprietor (permission issued by Paul Jones, University of London, London, UK, on August 28 2012).

### Pulmonary function testing

Pulmonary function was characterized using whole-body plethysmography. This enabled the assessment of residual volume and related measures such as functional reserve capacity and R_aw_. Whole-body plethysmography was performed according to the ATS/ERS criteria [30–32] using Piston whole-body plethysmograph (PDT-111/p, Piston Medical, Hungary) equipped with automatic BTPS correction for cabin temperature, humidity and pressure as well as full automatic calibration and leakage test. Patients were instructed to take their medication as usual even on the morning of their examination (so plethysmography was performed while patients were on asthma control therapy). Best of three technically sound maneuvers was selected for each participant. For resistance curves, at least two separate and technically appropriate measurements were performed (each measurement consists recordings of at least 5 resistance loops) and results were accepted only if they were the same for both measurements. Pulmonary function data as well as percent predicted of normal reference values were obtained using algorithms supplied by the manufacturer. The following parameters were included in the statistical analysis: R_aw_ (kPa s/L), G_aw_ (= 1/R_aw_), FEV1% pred, FVC % pred, FEV1/FVC, FEF25–75% % pred, RV% pred, TLC% pred, RV/TLC % pred, IC/TLC, IVC% pred, FEV1/IVC% pred, TGV% pred, PEF% pred and MEF50% % pred. Every patient underwent pulmonary function testing (n = 154).

### Blood samples

Blood samples were drawn in the morning of the examination after an overnight fast. Routine laboratory investigations were performed in line with the standard clinical practice at the Department of Laboratory Medicine (University of Debrecen). Accordingly, serum or plasma samples were used to determine parameters descriptive of carbohydrate (glucose, insulin, hemoglobin A1c (Hga1c)) and lipid homeostasis (total cholesterol, triglyceride, LDL-cholesterol, HDL-cholesterol, Lp(a), apoA1, apoB), of function of kidney (GFR, urea, creatinine), liver (GOT, GPT, γGT) and muscles (CK, LDH), and of inflammation (C-reactive protein (CRP), procalcitonin, fibrinogen). CRP was dichotomized as high vs. normal using the cutoff of 4.6 and 5.2 mg/L for female and male patients, respectively. HOMA index was calculated as described previously [6]. Other parameters related to l-arginine homeostasis were also assessed (folic acid, vitamin B12, urea). Serum samples used for the determination of l-arginine, ADMA and symmetrical dimethylarginine (SDMA) were frozen within 60 min and stored at − 80 °C until further analysis.

### Determination of arginine, ADMA and SDMA

Arginine and its dimethylated derivatives were measured as previously [5, 6]. Briefly: serum samples (250 μL) were mixed with 50 μL l-homoarginine hydrochloride (Sigma, Steinheim, Germany) as internal standard (1000 μmol/L) and 700 μL borate buffer (pH 9.00), then solutions were passed through the solid-phase extraction cartridges (OASIS MCX 3 cc) using a 12-column manifold (J. T. Baker, Philipsburg, NJ). After washing, arginine derivatives were eluted with 1 mL ammonia–water–methanol solution (10/40/50, v/v/v) (Reanal, Budapest, Hungary; Scharlau, Sentmenat, Spain). The solvent was evaporated in vacuum to dryness at 60 °C, then dissolved in 200 μL deionized water and used for derivatization.

The samples of 200 μL were mixed with 63 μL OPA/MPA (ortho-phthaldialdehyde/3-mercaptopropionic acid) reagent solution, incubated at 22 °C for 10 min, and then cooled down to 5 °C. Samples of 10 μL were injected into the chromatographic system consisting of a Waters 2695 Separations Module equipped with thermostable autosampler (5 °C) and column module (35 °C), a Waters 2745 Fluorescent detector (Waters Milford, MA, USA) and with C-18 (4.6 × 150 mm, 3.5 μm) column. Gradient elution at a flow rate of 1 mL/min was applied using mobile phase A (20 mmol/L (NH_4_)_2_CO_3_ in water, pH adjusted 7.50 ± 0.05) and mobile phase B (acetonitrile). The gradient condition was as follows: 0–13 min: 91% A and 9% B; 13–15 min: linear change to 70% A and 30% B and hold this setting for additional 5 min; 20–20.1 min: linear change to 91% A and 9% B and hold until 25 min.

Analytes were detected at λ_ex_ = 337 nm, λ_em_ = 520 nm was used for arginine and homoarginine and λ_em_ = 454 nm for ADMA and SDMA. Every patient had their serum sample analyzed (n = 154).

### St. George’s Respiratory questionnaire (SGRQ)

The validated Hungarian version of SGRQ was delivered according to the SGRQ manual supplied by the copyright holder [33]. This version is validated for a 1-month recall period related to the patients’ recollection of their symptoms. SGRQ was used to determine health impairment in patients by providing three component scores and a total score. The Symptoms score characterizes the effect, frequency and severity of respiratory symptoms over the preceding 1 month. It is reflective of the patients’ perception of their recent respiratory problems. The Activity score quantifies disturbances in daily physical activity caused to patients, whereas the Impacts score covers a wide range of disturbances related to psycho-social function. The Total score summarizes the significance of the disease on overall health status. Scores are expressed as a percentage with 100 representing the worst possible health status and 0 indicating the best one. Differences in scores were considered clinically meaningful if they exceeded 4 percent points [34]. Every patient filled out the questionnaire (n = 154) by means of supervised self-administration. Data entry was performed by two independent raters. Each used the SGRQ calculator, and coded all positive responses as 1 and all negative responses as 0. Where data were missing, cells were left blank. Data entry guidelines were diligently followed. Inter-rater variability assessed by Spearman correlation was 0.976 (p < 0.001), 0.997 (p < 0.001), 0.998 (p < 0.001) and 0.998 (p < 0.001) for the Symptoms, Activity, Impacts and Total scores of SGRQ, respectively. The mean of scores obtained by the independent raters was used for statistical analysis.

In addition, answers to Question 4 (Over the last 4 weeks, I have had attacks of wheezing) was used to assess the level of symptom control of asthma patients. Answers to this question were dichotomized as follows. Patients were considered well-controlled if they responded with yes to the options “not at all/only with chest infections”. Dichotomized response to Question 4 was used for stratification of data to allow further analysis of the final multiple regression model.

### Statistical analysis

Normality of distribution for continuous variables was checked with the Shapiro–Wilk test. If distribution was normal, Student’s t test was used for the comparison of two data sets, if not, Mann–Whitney U test was performed. Frequencies were compared with Pearson’s χ^2^ test.

Demographic, anthropometric, anamnestic, laboratory and SGRQ data were compared regarding the lower or higher extent of airflow limitation using R_aw_ values below 0.22 kPa s/L as cutoff. (Please note that this cutoff is considered as the cutoff for normal by the manufacturer of the plethysmograph used as well as by others [35], but it is coincidentally equivalent to the median value for Raw in our sample). Furthermore, demographic, anthropometric, pulmonary function and SGRQ data were compared in terms of the presence (well-controlled) or absence (not well-controlled) of adequate symptom control indicated by the response to Question 4 of SGRQ.

The correlation of R_aw_ and ADMA was established using Spearman’s correlation (because data sets did not follow Gaussian distributions). In order to account for potential confounders, multiple regression modeling was carried out. This procedure was initiated by assessing normality of each variable. Values of CK, HDL-cholesterol, Apo B, B12 vitamin, folate, sTSH, ADMA, SDMA, l-arginine, R_aw_ and G_aw_ were log-transformed, furthermore reciprocal of urea and reciprocal of square of glucose concentration were computed to ensure Gaussian distribution of variables prior to linear regression. Simple linear regression analysis was performed with possible determinants of R_aw_ and ADMA including traditional confounding factors (age, gender, height, disease duration in years) and indices descriptive of pulmonary function (mentioned above). Missing data were omitted. Furthermore, laboratory parameters descriptive of carbohydrate, lipid and arginine homeostasis, hepatic, kidney and skeletal muscle function as well as inflammation were assessed. After univariate testing, age and gender (as a priori variables) as well as all significant regressors were introduced into a multiple linear regression model to further quantify the relationship between airflow limitation (characterized by R_aw_ as the outcome variable) and serum ADMA concentration. (Inclusion of the defined daily dose of corticosteroids into the initial model should be noted.) Variables were entered in the model simultaneously, and then factors not significantly contributing to the model were deleted (eventually, the final model contained all variables identified a priori, FEF25-75% % pred, and the Activity and Total scores of SGRQ). In addition, the final model was stratified with respect to the presence or absence of asthma control indicated by responses to Question 4 of SGRQ. Heteroskedasticity of the model was assessed with Cook-Weisberg test.

Statistical analysis was performed with Stata 13.0 software (Stata Corporation). Values are given as mean ± SD or medians (with interquartile ranges: IQR), excepting regression coefficients which are presented with their 95% confidence intervals.

## Results

### Patients

The treatment history of the 154 asthma patients included in our study were as follows: 4 patients were treatment naïve at the time of inclusion; 3 patients received a fixed combination of ipratropium with fenoterol; 45 patients were treated with short-acting beta agonists (43 of them with an inhaled corticosteroid); 146 patients received inhaled corticosteroid as a mono-component preparation (n = 18) or in fixed combination with long-acting and/or short-acting beta agonists (n = 128). Other medications included inhalational use of anticholinergic agents (n = 38), oral use of methylxanthines (n = 16), montelukast (n = 35) and omalizumab subcutaneously (n = 8). In summary, most asthma patients received inhaled corticosteroids (and if not, it was always due to co-morbidities rendering the risks related to corticosteroid therapy higher than the accrued benefits).

### Comparison of patients with lower and higher airway resistance

Dichotomization of the patient population by R_aw_ (using the cutoff of R_aw_ < 0.22 for lower airway resistance) yielded two patient populations (n = 77 and n = 77) being homogenous with respect to most of parameters investigated (Table 1). Nevertheless, distribution of men and women differed as 42 and 28 men were present in the group without and with airflow limitation, respectively (p = 0.023). In addition, the patient’s height was slightly but statistically significantly smaller in the group showing increased R_aw_, while duration of asthma, serum ADMA level and all SGRQ components and the total scores were significantly greater in the group with elevated R_aw_. Dyslipidemia was also more frequent among patients with higher R_aw_ (Table 1).

**Table 1:** Some characteristics of the whole population of bronchial asthma patients (n = 154) and of its two strata dichotomized according to lower (n = 77) or higher (n = 77) airway resistance (R_aw_)

Parameters	Whole population	Lower R_aw_	Higher R_aw_	p
Age (years)	49.00 (36.00–58.00)	44.00 (36.00–57.00)	52.00 (41.00–59.00)	0.086
Gender (f/m)	84/70	35/42	49/28	*0.023*
Smoker (n/y)	134/20	66/11	68/9	0.632
Smoking (pack-years)	0.00 (0.00–3.88)	0.00 (0.00–4.00)	0.00 (0.00–3.75)	0.987
Diabetes (n/y)	147/7	76/1	71/6	0.053
Dyslipidemia (n/y)	108/46	63/14	45/32	*0.002*
RR systolic (mmHg)	132.10 ± 15.08	132.93 ± 15.46	131.23 ± 14.73	0.487
RR diastolic (mmHg)	84.76 ± 10.70	85.80 ± 9.75	83.73 ± 11.53	0.231
Hypertension (n/y)	95/59	53/24	42/35	0.068
Disease duration (years)	15.00 (10.00–20.00)	14.00 (8.00–19.00)	16.00 (12.00–21.00)	*0.032*
Waist (cm)	96.49 ± 12.87	96.02 ± 13.51	96.97 ± 12.28	0.654
Weight (kg)	75.00 (65.00–88.00)	76.00 (66.00–89.50)	75.00 (65.00–87.00)	0.632
Height (m)	1.68 ± 0.10	1.70 ± 0.10	1.66 ± 0.09	*0.004*
BMI (kg/m^2^)	27.16 ± 4.48	26.61 ± 4.56	27.72 ± 4.36	0.127
ADMA (µmol/L)	0.54 (0.44–0.67)	0.53 (0.40–0.65)	0.57 (0.47–0.71)	*0.034*
SDMA (µmol/L)	0.45 (0.38–0.53)	0.45 (0.36–0.52)	0.47 (0.41–0.55)	0.080
l-arginine (µmol/L)	103.17 (90.24–125.84)	101.32 (87.65–117.68)	104.58 (91.70–129.90)	0.209
B12 (pmol/L)	322.25 (237.30–398.10)	321.00 (228.80–390.70)	323.50 (239.20–401.10)	0.432
Folate (nmol/L)	19.00 (15.19–23.99)	19.50 (14.76–24.15)	18.91 (16.00–23.16)	0.779
Urea (mmol/L)	4.50 (3.90–5.50)	4.30 (3.90–5.30)	4.60 (3.90–5.55)	0.667
Creatinine (µmol/L)	69.00 (59.00–80.00)	70.00 (60.00–79.00)	69.00 (57.00–80.00)	0.762
GFR (ml/min/1.73 m^2^)	91.00 (85.00–91.00)	91.00 (87.00–91.00)	91.00 (85.00–91.00)	0.469
GOT (U/L)	20.00 (16.00–25.00)	21.00 (17.00–24.00)	19.00 (16.00–25.50)	0.314
GPT (U/L)	19.00 (15.00–28.00)	19.00 (15.00–27.00)	18.00 (14.00–29.00)	0.314
γGT (U/L)	23.00 (16.00–34.00)	23.00 (16.00–32.00)	22.50 (15.50–34.50)	0.920
CK (U/L)	110.00 (81.00–158.00)	114.00 (88.00–167.00)	106.50 (75.50–152.00)	0.090
LDH (U/L)	196.00 (180.00–223.00)	195.00 (180.00–224.00)	197.50 (179.00–222.00)	0.775
Glucose (mmol/L)	5.00 (4.30–5.50)	5.00 (4.20–5.40)	5.00 (4.50–5.50)	0.388
Insulin (mU/L)	9.05 (6.25–17.35)	9.00 (6.40–16.50)	9.50 (6.00–17.70)	0.569
HgA1C (%)	5.40 (5.00–5.80)	5.40 (5.00–5.70)	5.50 (5.00–5.80)	0.266
HOMA	2.01 (1.28–4.11)	1.99 (1.24–3.38)	2.19 (1.35–4.25)	0.405
Cholesterol (mmol/L)	5.32 ± 1.20	5.19 ± 1.04	5.44 ± 1.33	0.208
LDL-C (mmol/L)	3.18 ± 0.94	3.08 ± 0.86	3.27 ± 1.02	0.230
HDL-C (mmol/L)	1.40 (1.20–1.75)	1.40 (1.20–1.70)	1.40 (1.20–1.90)	0.429
Apo-A1 (g/L)	1.58 ± 0.29	1.56 ± 0.25	1.60 ± 0.32	0.393
ApoB (g/L)	0.99 (0.85–1.18)	0.95 (0.85–1.08)	1.00 (0.85–1.22)	0.320
Lp(a) (mg/L)	121.00 (55.00–352.00)	117.00 (52.00–346.00)	132.00 (56.00–364.00)	0.913
TG (mmol/L)	1.30 (1.00–2.00)	1.30 (0.90–2.00)	1.45 (1.00–1.95)	0.969
CRP (high/low)	28/125	13/64	15/61	0.648
Fibrinogen (g/L)	3.35 ± 0.64	3.30 ± 0.55	3.39 ± 0.72	0.422
Procalcitonin (µg/L)	0.00 (0.00–0.00)	0.00 (0.00–0.00)	0.00 (0.00–0.00)	0.317
SGRQ Symptoms score	29.97 (13.47–52.33)	23.04 (11.01–43.24)	33.83 (14.56–55.63)	*0.034*
SGRQ Impacts score	24.60 (10.74–40.01)	16.10 (7.39–34.92)	30.99 (20.50–48.05)	*<* *0.001*
SGRQ Activity score	47.68 (29.49–60.25)	35.47 (17.31–54.32)	55.62 (41.70–66.18)	*<* *0.001*
SGRQ Total score	32.75 (17.52–48.73)	25.26 (13.90–41.00)	37.93 (29.53–54.01)	*<* *0.001*

The cutoff for R_aw_ was 0.22 kPa s/L, with < 0.22 and ≥ 0.22 kPa s/L indicating lower (n = 77) and higher (n = 77) airway resistance, respectively. Data are presented as mean ± SD or median (interquartile range) unless otherwise stated

*SGRQ* St. George’s Respiratory questionnaire

Differences between the two groups were considered significant at p < 0.05 (indicated in italics)

### Comparison of the well-controlled and not well-controlled patients

Dichotomy of patients with regards to the response to Question 4 of SGRQ provided a well-controlled (n = 123) and a not well-controlled (n = 31) stratum (Table 2). Demographic and anthropometric characteristics, furthermore static pulmonary function parameters (volumes) did not differ significantly when compared these two groups (with the exception of height that was slightly but significantly smaller in the not well-controlled group, similarly to the group with elevated R_aw_). Dynamic lung function parameters characterizing the flow in the airways were significantly smaller in the not well-controlled group, while there was no significant difference between the two groups with regard to objective measures of airway obstruction (e.g. R_aw_ and its G_aw_). All SGRQ scores were significantly higher in the not well-controlled group (indicating greater health impairment and poorer quality of life) (Table 2).

**Table 2:** Main characteristics of the whole population of bronchial asthma patients (n = 154) and of its two strata divided on the basis of the response to Question 4 of SGRQ, i.e. well-controlled (n = 123) and not well-controlled (n = 31) groups

Parameters	Whole population	Well-controlled	Not well-controlled	p
Demographic and anthropometric parameters				
Age (years)	49.00 (36.00–58.00)	48.00 (36.00–59.00)	52.00 (40.00–56.00)	0.442
Gender (f/m)	84/70	63/60	21/10	0.099
Height (m)	1.68 ± 0.098	1.69 ± 0.097	1.65 ± 0.099	*0.034*
Weight (kg)	75.00 (65.00–88.00)	76.00 (67.00–88.00)	72.00 (62.00–87.00)	0.278
Lung function parameters				
FVC% pred	92.55 ± 13.80	93.45 ± 13.60	89.00 ± 14.25	0.101
IVC% pred	97.50 (87.00–106.00)	98.00 (88.00–106.00)	97.00 (81.00–102.00)	0.267
TLC% pred	108.65 ± 13.78	108.25 ± 14.27	110.26 ± 11.75	0.470
TGV% pred	125.25 ± 22.11	124.62 ± 22.18	127.77 ± 21.99	0.479
RV% pred	132.50 (117.00–157.00)	132.00 (117.00–152.00)	143.00 (114.00–175.00)	0.266
RV/TLC % pred	123.84 ± 20.06	122.41 ± 19.84	129.48 ± 20.24	0.079
FEV1% pred	86.10 ± 15.07	87.99 ± 13.97	78.58 ± 17.08	*0.002*
PEF% pred	75.14 ± 18.21	78.80 ± 16.12	60.61 ± 18.98	*<* *0.001*
FEF25–75% % pred	66.54 ± 22.22	69.87 ± 21.58	53.32 ± 19.98	*<* *0.001*
MEF50% % pred	68.81 ± 24.94	72.36 ± 23.85	54.74 ± 24.58	*<* *0.001*
FEV1/IVC% pred	90.92 ± 10.51	92.41 ± 10.23	85.00 ± 9.61	*<* *0.001*
FEV1/FVC	0.77 (0.73–0.83)	0.79 (0.74–0.84)	0.74 (0.68–0.78)	*0.002*
R_aw_ (kPa s/L)	0.22 (0.18–0.29)	0.21 (0.17–0.28)	0.25 (0.19–0.31)	0.152
G_aw_ (L/kPa s)	4.67 (3.44–5.55)	4.78 (3.55–5.73)	3.94 (3.2–5.35)	0.149
SGRQ				
Symptoms score	29.97 (13.47–52.33)	22.44 (11.01–37.28)	64.49 (51.75–74.89)	*<* *0.001*
Impacts score	24.60 (10.74–40.01)	20.50 (8.58–34.92)	42.59 (32.10–54.24)	*<* *0.001*
Activity score	47.68 (29.49–60.25)	41.76 (23.33–59.45)	59.45 (53.01–72.85)	*<* *0.001*
Total score	32.75 (17.52–48.73)	28.35 (16.22–41.55)	53.71 (41.22–60.93)	*<* *0.001*

Data are presented as mean ± SD or median (interquartile range) unless otherwise stated

*SGRQ* St. George’s Respiratory questionnaire

Differences between the two groups were considered significant at p < 0.05 (indicated in italics)

### Significant predictors of airway resistance and serum ADMA concentration

**Fig. 2:**
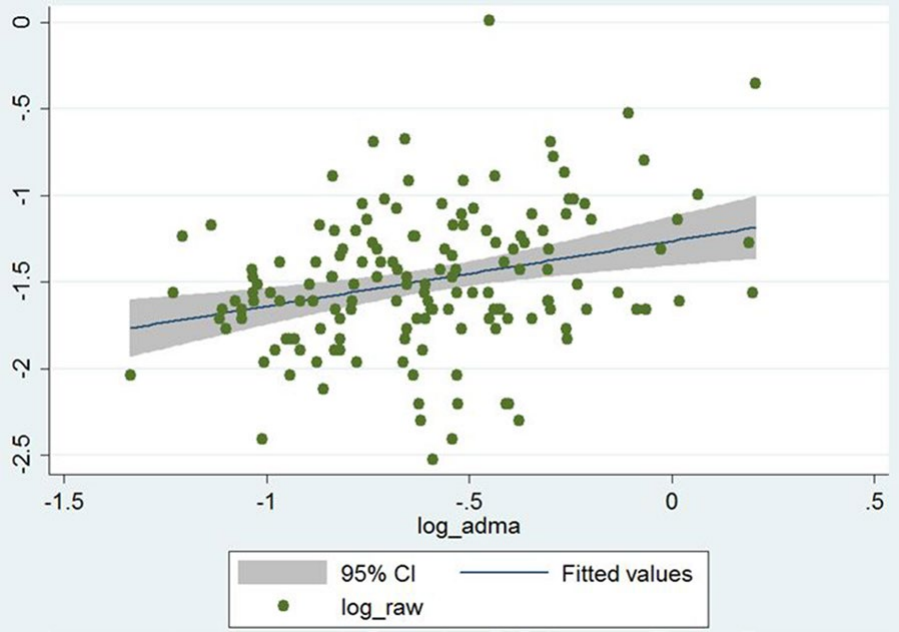
Correlation of airway resistance (R_aw_) and asymmetric dimethylarginine (ADMA) serum levels in the whole data set (n = 154). The grey zone indicates the 95% confidence interval, while the blue line (in it) shows the fitted values of log ADMA and log R_aw_ data pairs

The (log transformed) R_aw_ showed positive correlation with (log transformed) ADMA upon the analysis of the whole data set (Spearman correlation coefficient: 0.27, p < 0.001) (Fig. 2) and of data obtained from the well-controlled asthma stratum (Spearman correlation coefficient: 0.30, p < 0.001). In contrast, no statistically significant correlation was found in the not well-controlled asthma stratum (Spearman correlation coefficient: 0.12, p = 0.51).

**Fig. 3:**
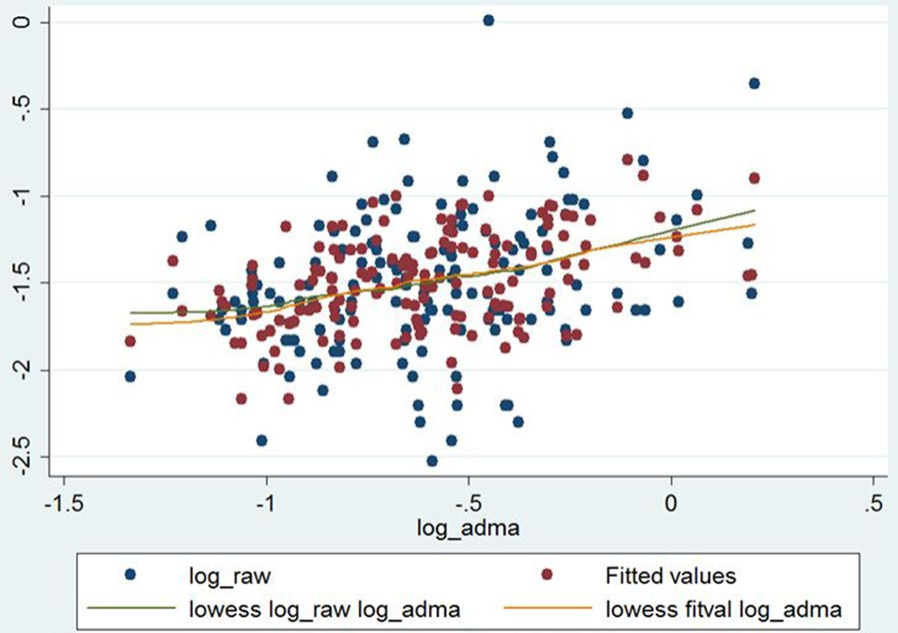
The model of correlation of airway resistance (R_aw_) and asymmetric dimethylarginine (ADMA) serum concentration. The blue dots indicate the raw (i.e. original) data, while the red dots indicate the fitted values obtained by multiple linear regression. The green and orange lines indicate the fitted curves for raw data and for data provided by multiple regression. The fitted curves were obtained by locally weighted scatterplot smoothing (lowess)

Consistently, the simple linear regression (used to identify which parameters determine R_aw_ and serum ADMA concentration) has proved that R_aw_ and ADMA are mutually significant predictors for each other (Table 3). It is interesting to note that FEV1% predicted, a parameter commonly used in clinical practice to characterize airway function showed significant linear association with (log) ADMA (β: − 0.0035, CI − 0.0067, − 0.00,020; p = 0.01). The positive association seen between (log) R_aw_ and (log) ADMA remained statistically significant after multiple linear regression (β: 0.22, CI: 0.054, 0.383; p = 0.01), even after adjusting for all significant predictors and determinants identified in advance (Table 3). The positive association between (log) R_aw_ and (log) ADMA became even more pronounced in the stratum of well-controlled asthma patients (β: 0.25, CI: 0.08, 0.41; p = 0.005), while there was a weak, statistically not significant association in the not well-controlled stratum (β: 0.14, CI: − 0.40, 0.67; p = 0.60). The final model and its stratified models showed no heteroskedasticity (p = 0.57, p = 0.78 and p = 0.19 for the full model, well-controlled and not well-controlled strata, respectively). The final model showed good fit reflected by the Cook-Weisberg test (p = 0.57) and by locally weighted scatterplot smoothing (Fig. 3).

**Table 3:** Significant (and two almost significant) predictors of asymmetric dimethylarginine (ADMA) serum level and airway resistance (R_aw_) determined with simple and multiple (for R_aw_ only) linear regression for the whole population of bronchial asthma patients (n = 154)

Parameter	Coefficient (95% CI)	p
Simple linear regression of ADMA (log transformed)		
Height	− 0.75 (− 1.25, − 0.26)	*0.003*
Disease duration	0.0061 (0.00062, 0.012)	*0.029*
log l-arginine	0.27 (0.083, 0.45)	*0.005*
log SDMA	0.77 (0.62, 0.93)	*<* *0.001*
log TG	0.091 (0.0022, 0.18)	*0.045*
GFR (normal/low)	0.12 (0.022, 0.23)	*0.018*
FVC% pred	− 0.0037 (− 0.0072, 0.00010)	*0.044*
FEV1% pred	− 0.0035 (− 0.0067, − 0.00020)	*0.038*
FEF25–75% % pred	− 0.0022 (− 0.0044, 0.000013)	0.051
log R_aw_	0.22 (0.10, 0.34)	*<* *0.001*
log G_aw_	− 0.22 (− 0.33, − 0.10)	*<* *0.001*
Simple linear regression of R_aw_ (log transformed)		
Age	0.0046 (0.00019, 0.0090)	*0.041*
Gender	− 0.20 (− 0.33, − 0.075)	*0.002*
Height	− 1.22 (− 1.86, − 0.58)	*<* *0.001*
Disease duration	0.0077 (0.00051, 0.015)	*0.036*
Dyslipidemia	0.18 (0.043, 0.32)	*0.011*
Hypertension	0.19 (0.059, 0.32)	*0.005*
BMI	0.014 − 0.000066, 0.029)	0.051
Albumin	− 0.028 (− 0.051, − 0.0052)	*0.017*
log ADMA	0.38 (0.17, 0.58)	*<* *0.001*
log SDMA	0.29 (0.031, 0.54)	*0.028*
FVC% pred	− 0.010 (− 0.015, − 0.0059)	*<* *0.001*
FEV1% pred	− 0.14 (− 0.017, − 0.0099)	*<* *0.001*
FEV1/FVC	− 0.018 (− 0.0263, − 0.011)	*<* *0.001*
FEF25–75% % pred	− 0.15 (− 0.24, − 0.062)	*0.001*
RV% pred	0.0022 (0.00033, 0.0042)	*0.022*
RV/TLC% pred	0.0068 (0.0037, 0.0098)	*<* *0.001*
IC/TLC	− 1.22 (− 1.95, − 0.45)	*0.001*
SGRQ activity score	0.0071 (0.0044, 0.0097)	*<* *0.001*
SGRQ impacts score	0.0044 (0.00094, 0.0079)	*0.013*
SGRQ total score	0.0059 (0.0025, 0.0093)	*0.001*
Multiple linear regression of R_aw_ (log transformed)		
log ADMA	0.22 (0.054, 0.383)	*0.01*
FEF25–75% % pred	− 0.009 (− 0.01, − 0.006)	*<* *0.001*
SGRQ activity score	0.009 (0.004, 0.014)	*<* *0.001*
SGRQ total score	− 0.007 (− 0.012, − 0.001)	*0.019*

Statistically significant p values are indicated in italic (p < 0.05)

Regression coefficient values are presented with their 95% confidence limits

Significant parameters provided by the simple regression (together with the relevant a priori identified parameters) served as an initial model for the multiple regression analysis

In addition, the final model (for R_aw_) indicated a negative association with FEF25–75% % pred, a lung function parameter descriptive of small airway dysfunction and a positive correlation with the Activity score of SGRQ reflective of the disturbance the patient suffers with respect to daily physical activity. Surprisingly, in the final model, R_aw_ is negatively associated with the global effect disease has on the patient’s well-being (reflected by the Total score of SGRQ) (Table 3).

Further assessment of the final model exhibited that (log) R_aw_ showed good correlation with FEF25–75% % pred in the whole, well-controlled and not well-controlled strata (Spearman correlation coefficient: − 0.53, − 0.54 and − 0.39, p < 0.001, p < 0.001 and p = 0.027, respectively). Consistently, there was a strong, significant positive correlation between (log) G_aw_ and FEF25–75% % pred in the whole sample, in the well-controlled and in the not well-controlled stratum too (Spearman correlation coefficient: 0.53, 0.54 and 0.39, p < 0.001, p < 0.001 and p = 0.031, respectively). In addition, total score of SGRQ showed a significant negative correlation with FEV1% pred (Spearman correlation coefficient: − 0.33, p < 0.001), which correlation also remained significant in both the well-controlled and not well-controlled strata (Spearman correlation coefficient: − 0.24 and − 0.36, p = 0.007 and p = 0.049, respectively). (Other parameters indicative of small airway disease such as RV% pred and RV/TLC% pred [36, 37], which were also included in the original model, did not contribute significantly to the model thus they were finally omitted.)

## Discussion

The main finding of the current study is that serum ADMA shows significant positive correlation with airway limitation characterized by R_aw_ in adult asthma patients receiving asthma controller therapy (including, for most patients, an inhaled corticosteroid). This relationship remained significant even after adjusting for potential confounders. Furthermore, this positive association was more pronounced when the analysis was restricted to patients attaining a high level of asthma control. Upon evaluating the inflammatory status of our patient cohort, we found that general markers of inflammation (CRP, procalcitonin and fibrinogen) are in the normal range and show no significant difference with respect to airflow limitation (R_aw_). This lack of evidence for systemic inflammation could be due to the fact that these patients all received control-based asthma therapy at the time of our investigations (94% of them (n = 146) were on inhaled corticosteroid therapy).

These findings have several implications regarding l-arginine-NO homeostasis including the interaction between ADMA and distinct NOS isoforms. Prior reports demonstrated that use of even low-dose corticosteroids is able to inhibit the activation of NF-κB and the subsequent iNOS synthesis [12]. Moreover, glucocorticoids can decrease systemic inflammatory signals needed for inducing iNOS transcription [8]. Considering these effects together with the observation that ADMA seems to preferentially inhibit constitutive NO synthases [38], we postulate that, in our cohort (mainly in the well-controlled stratum), iNOS is not or minimally induced and thus its inhibition by ADMA is marginal. This means that the potential beneficial effect of ADMA conferred by inhibition of iNOS may be insignificant. Therefore, we suggest that, in our sample, inhibition of NOS is only evident with respect to the constitutive isoforms. As such, elevated levels of ADMA may elicit deleterious effects by decreasing NO produced by the constitutive NOS isoforms. Furthermore, ADMA, by uncoupling NOS, could lead to increased reactive oxygen and nitrogen species formation in airway epithelial cells [38]. Starting from the observation that ADMA enhanced arginase activity leading to collagen production [20], a reversible fibrosis provoked by ADMA (Fig. 1) may also contribute to the increase of R_aw_. This concept is further supported by the weaker association seen between R_aw_ and serum ADMA levels (indicated by the lower value of the regression coefficient and lack of statistical significance). Given the fact that presence of wheezing is possibly indicative of airway inflammation (being present regardless of the medication used), it may be presumed that these inflammatory processes induce iNOS. It would follow that inhibition of iNOS by ADMA in these patients (e.g. in the not well-controlled stratum) hence would counteract the deleterious effects exerted by iNOS and would manifest in a lower increase of R_aw_.

The fact that significant difference was only evidenced for dynamic lung function parameters when patients were compared with respect to their level of symptom control (Table 2) may be due to fact that flow parameters (as well as the Tiffeneau index) compile information regarding airway resistance and respiratory effort. The latter is influenced by elastic recoil of the lung, respiratory muscle strength and stiffness of the chest. It is highly probable that those patients who experienced wheezing over the past 4 weeks are in a poorer general physical condition, further reflected by the significantly and clinically meaningful differences in the Symptoms, Activity and Impacts component scores as well as the totals score for SGRQ. So, it seems probable that the significant difference seen with respect to flow parameters is due to the difference in general physical state and consequent muscle strength of these two groups. This difference is absent in objective measures of airflow limitation, e.g. R_aw_ and G_aw_ as these measures are not influenced by respiratory effort.

Previously we have proposed that, in the range of normal concentrations (0.35–1.0 μmol/L), ADMA confers protection against atherosclerosis [6], a lesion where iNOS is induced, by causing a more pronounced inhibition on iNOS due to the lower EC_50_ value of ADMA for iNOS than that for eNOS [39, 40]. Although, in the present study, serum ADMA levels were also in the normal range, this beneficial effect of ADMA was not seen possibly due to the inhibitory effect inhaled corticosteroids have on airway iNOS expression (which makes inhibition of iNOS by ADMA insignificant in asthma patients receiving corticosteroid therapy).

In general, few reports are available regarding the systemic level of ADMA in pediatric and adult asthma patients. Our ADMA values are comparable with results of others. In a recent study, systemic ADMA levels of 0.37 μmol/L (IQR: 0.29, 0.59) and 0.48 μmol/L (IQR: 0.35, 0.7) were reported for adult patients suffering from early- and late-onset asthma, respectively [22]. Upon assessment of children with asthma, a group found serum ADMA levels corresponding to 0.53 μmol/L (CI: 0.47, 0.6) [41], while yet another group reported mean plasma ADMA concentration of 0.58 ± 0.05 μmol/L [23]. Others described considerably higher circulating levels of ADMA in children suffering from asthma (0.92 ± 0.20 μmol/L), however this showed no significant difference when compared to healthy controls also included in that study (0.91 ± 0.23 μmol/L, p = 0.88) [42].

Our finding that the final model describing the relationship between R_aw_ and ADMA includes FEF25–75% % pred (Table 3) could be interpreted in view of the well-established notion that asthma compromises the function of the small airways [43]. This is especially true for airways with internal diameter lower than 2 mm [44]. In fact, small airways were implicated to be one of the chief sites of airflow limitation [45]. In line with these observations, we have found that FEF25–75% % pred shows a strong negative correlation with R_aw_ implying the contribution of small airways to airflow limitation (accordingly, G_aw_, the reciprocal of R_aw_, exhibits a significant positive correlation with FEF25–75% % pred). The fact that this strong correlation proved to be significant irrespective of the level of asthma control achieved is interesting if one considers that targeting distal airways by means of an inhalational therapy remains a challenge [43]. Our finding that inflammation of small airways (reflected by decreased FEF25–75% % pred) parallels that of larger airways (indicated by increased R_aw_) emphasizes that special attention must be paid to optimal delivery of inhalational medication in daily clinical practice to allow for sufficient relief of airflow limitation stemming from the distal airways.

The third set of parameters that contribute significantly to the explanation of the relationship between R_aw_ and ADMA is related to the health impairment reported by asthma patients of our cohort. In agreement with prior studies [46], we have found a significant negative correlation between the Total score of SGRQ and FEV1% pred. Furthermore, we found that Activity score shows significant positive correlation with R_aw_, indicating that airway limitation is associated with loss of quality of life conferred by disturbance of physical activity. The negative association seen between the Impacts score and R_aw_ in our cohort may be due to the correction of overrepresentation of the influence of Activity score due to the fact that it is included in the Total score as well (so, it might be an inherent feature of SGRQ). The clinically meaningful difference of the three components as well as the Total score compared between groups of lower and higher R_aw_ values (Table 1) further emphasizes the deleterious effect of airway limitation on the quality of life of patients.

A limitation of the present study is the lack of direct evidence for the activity of distinct NOS isoforms (as specimens related to the different compartments). Thus, we only presume a suppressed iNOS activity starting from the absence of elevated inflammatory markers in the serum that makes the explanation of our findings somewhat speculative. Further limitation of the study relates to the lack of characterization of oxidative and nitrosative stress by means of determining stable end products (nitrite and nitrate). In addition, it must be acknowledged that Raw was not expressed as percent predicted (described by [47]) due to technical limitations, hence this measure was not adjusted for weight and height. Nevertheless, height and BMI identified as significant regressors for Raw and/or (log)ADMA (by the simple linear regression (Table 3)) were included in the initial multiple model. However, both of these parameters were omitted due to their lack of significant contribution to the final model. FEF25–75% % pred should be interpreted with caution because of difficulties with reproducibility previously reported by others [36]. Finally, it should be mentioned that our analysis does not address the timing of the measurements in relationship to the administration of bronchodilators or other medications, thus although patients were examined while being on maintenance therapy, timing of the medication consumption may induce a variability.

## Conclusions

To the best of our knowledge, this is the first study in which the relationship was investigated between serum ADMA level and R_aw_, an objective functional parameter characteristic of airflow limitation in asthma. The strong positive correlation between ADMA and R_aw_ in a multiple linear regression model may indicate that ADMA contributes to the development of bronchoconstriction in asthma patients receiving control-based asthma therapy. This is further emphasized by the fact that this correlation became even stronger when the analysis was limited to the stratum of well-controlled patients. Based on the correlation between ADMA and R_aw_, we shed light on the potential importance of ADMA in the pathomechanism of asthma. In addition, our results further demonstrate that R_aw_ is a valuable parameter in the assessment of airflow limitation in asthma patients.

## Abbreviations

ACOS: asthma-COPD overlap syndrome
ADMA: asymmetric dimethylarginine
apoA1: apolipoprotein A1
apoB: apolipoprotein B
AR: allergic rhinitis
ATS: American Thoracic Society
BTPS: body temperature and pressure saturated
CK: creatine kinase
COPD: chronic obstructive pulmonary disease
CRP: C-reactive protein
DDAH: dimethylargininase
EC_50_: agonist concentration producing half maximal effect
eNOS: endothelial NOS
ERS: European Respiratory Society
FEF25–75% % pred: forced expiratory flow between 25 and 75% of FVC, percent of predicted value
FEV1% pred: forced expiratory volume in 1 s, percent of predicted value
FEV1/FVC: ratio of FEV1 to FVC = FEV1% = Tiffeneau index
FEV1/IVC% pred: ratio of FEV1 to IVC, percent of predicted value
FVC% pred: forced vital capacity, percent of predicted value
G_aw_: airway conductance
GINA: Global Initiative for Asthma
GOT: glutamate-oxaloacetate transaminase
GPT: glutamate-pyruvate transaminase
HDL: high-density lipoprotein
HgA1c: hemoglobin A1c (glycated hemoglobin)
HOMA: homeostasis model assessment
HPLC: high-performance liquid chromatography
IC/TLC: ratio of inspiratory capacity to total lung capacity
iNOS: inducible NOS
IQR: interquartile range
IVC% pred: inspiratory vital capacity, percent of predicted value
LDH: lactate dehydrogenase
LDL: low-density lipoprotein
Lp(a): lipoprotein (a)
MEF50% % pred: maximal expiratory flow at 50% of FVC, percent of predicted value
MPA: 3-mercaptopropionic acid
NF-κB: nuclear factor kappa-light-chain-enhancer of activated B cells
nNOS: neuronal NOS
NO: nitric-oxide
NOHA: *N*-hydroxyarginine
NOS: nitric-oxide synthase
O^2−^: superoxide anion
ONOO^−^: peroxynitrite anion
OPA: ortho-phthalaldehyde
PEF% pred: peak expiratory flow, percent of predicted value
PRMT: protein methyltransferase
R_aw_: airway resistance
RV% pred: residual volume, percent of predicted value
RV/TLC% pred: ratio of RV to TLC, percent of predicted value
SD: standard deviation
SDMA: symmetrical dimethylarginine
SGRQ: St. George’s Respiratory questionnaire
STROBE: strengthening the reporting of observational studies in epidemiology
sTSH: thyroid-stimulating hormone determined in a sensitive manner
TGV% pred: thoracic gas volume, percent of predicted value
TLC% pred: total lung capacity, percent of predicted value
γGT: gamma-glutamyl transferase
